# Aldehyde accumulation in *Mycobacterium tuberculosis* with defective proteasomal degradation results in copper sensitivity

**DOI:** 10.1128/mbio.00363-23

**Published:** 2023-06-23

**Authors:** Gina Limón, Nora M. Samhadaneh, Alejandro Pironti, K. Heran Darwin

**Affiliations:** 1 Department of Microbiology, New York University Grossman School of Medicine, New York, New York, USA; 2 Antimicrobial-Resistant Pathogens Program, New York University Grossman School of Medicine, New York, New York, USA; 3 Microbial Computational Genomic Core Lab, New York University Grossman School of Medicine, New York, New York, USA; The Hebrew University of Jerusalem, Jerusalem, Rehobot, Israel

**Keywords:** *Mycobacterium tuberculosis*, copper, aldehyde, proteasome

## Abstract

*Mycobacterium tuberculosis* is a major human pathogen and the causative agent of tuberculosis disease. *M. tuberculosis* is able to persist in the face of host-derived antimicrobial molecules nitric oxide (NO) and copper (Cu). However, *M. tuberculosis* with defective proteasome activity is highly sensitive to NO and Cu, making the proteasome an attractive target for drug development. Previous work linked NO susceptibility with the accumulation of *para*-hydroxybenzaldehyde (*p*HBA) in *M. tuberculosis* mutants with defective proteasomal degradation. In this study, we found that *p*HBA accumulation was also responsible for Cu sensitivity in these strains. We showed that exogenous addition of *p*HBA to wild-type *M. tuberculosis* cultures sensitized bacteria to Cu to a degree similar to that of a proteasomal degradation mutant. We determined that *p*HBA reduced the production and function of critical Cu resistance proteins of the regulated in copper repressor (RicR) regulon. Furthermore, we extended these Cu-sensitizing effects to an aldehyde that *M. tuberculosis* may face within the macrophage. Collectively, this study is the first to mechanistically propose how aldehydes can render *M. tuberculosis* susceptible to an existing host defense and could support a broader role for aldehydes in controlling *M. tuberculosis* infections.

**IMPORTANCE:**

*M. tuberculosis* is a leading cause of death by a single infectious agent, causing 1.5 million deaths annually. An effective vaccine for *M. tuberculosis* infections is currently lacking, and prior infection does not typically provide robust immunity to subsequent infections. Nonetheless, immunocompetent humans can control *M. tuberculosis* infections for decades. For these reasons, a clear understanding of how mammalian immunity inhibits mycobacterial growth is warranted. In this study, we show aldehydes can increase *M. tuberculosis* susceptibility to copper, an established antibacterial metal used by immune cells to control *M. tuberculosis* and other microbes. Given that activated macrophages produce increased amounts of aldehydes during infection, we propose host-derived aldehydes may help control bacterial infections, making aldehydes a previously unappreciated antimicrobial defense.

## INTRODUCTION

*Mycobacterium tuberculosis* is a major human-exclusive pathogen and the causative agent of tuberculosis disease (TB). TB is responsible for more than 1 million deaths annually and was the deadliest infectious disease worldwide prior to the severe acute respiratory syndrome coronavirus 2 pandemic (https://www.who.int/news-room/fact-sheets/detail/tuberculosis). TB can be cured by lengthy treatments with multiple antibiotics; however, antibiotic-resistant strains of *M. tuberculosis* are increasingly prevalent. Thus, an improved understanding of *M. tuberculosis* pathogenesis and existing host responses to the bacteria are required to aid in the development of new treatment strategies.

Macrophages, a primary cell niche of *M. tuberculosis*, require the production of nitric oxide (NO) for robust resistance to various infections, in particular *M. tuberculosis* (1, 2). NO is a free radical that can form toxic reactive intermediates that damage macromolecules, including nucleic acids, proteins, and lipids [reviewed in (1)]. While the precise mechanism of NO-mediated toxicity to *M. tuberculosis* is unknown, *M. tuberculosis* requires a Pup-proteasome system (PPS) to resist NO (3). In the PPS, numerous proteins destined for degradation are post-translationally modified with Pup (prokaryotic ubiquitin-like protein) by the ligase proteasome accessory factor A (PafA) [reviewed in (4)]. Pup directly binds to a hexameric ATPase, mycobacterial proteasome ATPase (Mpa; ARC in non-mycobacteria), which unfolds pupylated proteins and delivers them into a proteasome core protease. In the absence of either Mpa or PafA, numerous proteins fail to be degraded. Importantly, when the PPS is nonfunctional in *M. tuberculosis*, the enzyme lonely guy (Log) accumulates (5). Log plays a role in the production of adenine-based hormones called cytokinins, which are required for normal growth and development in plants (6). *M. tuberculosis* is not found in plants and instead uses cytokinins to induce gene transcription that alters the mycobacterial cell envelope, a phenotype that is not linked to NO resistance (5, 7). In plants, cytokinins are enzymatically broken down into adenine and various aldehydes (8). While it is unknown how cytokinins are metabolized in *M. tuberculosis*, at least one cytokinin-associated aldehyde, *para*-hydroxybenzaldehyde (*p*HBA), measurably accumulates in a PPS mutant*,* and this aldehyde is sufficient to render wild-type (WT) *M. tuberculosis* sensitive to NO (5). However, the mechanistic link between aldehyde accumulation and NO sensitization of *M. tuberculosis* remains unknown.

In addition to NO, there is evidence that macrophages use copper (Cu) to defend against *M. tuberculosis* and other microbial infections (9 - 12). *M. tuberculosis* has two defined Cu-responsive systems to mitigate Cu toxicity: the copper-sensitive operon repressor (CsoR) operon (13, 14) and the regulated in copper repressor (RicR) regulon (15). CsoR and RicR are paralogues that regulate Cu resistance, although the RicR regulon appears to play a more substantial role in Cu resistance in mice (12, 15 - 18). When Cu concentrations are low, RicR binds to the promoters of five loci, including its own promoter, blocking their expression. When Cu levels increase, Cu binds to RicR, preventing it from associating with DNA and allowing gene expression. Two RicR-regulated gene products, MmcO, a multicopper oxidase, and MymT, a Cu metallothionein, confer resistance to Cu; deletion of either *mmcO* or *mymT* results in Cu hypersusceptibility *in vitro* (16, 18, 19). While the deletion of any single RicR-regulated gene does not attenuate *M. tuberculosis* growth in C57BL6/J mice, the production of a “Cu-blind” RicR protein that constitutively represses expression of the entire regulon results in highly Cu-sensitive bacteria that are attenuated for growth *in vivo* (18).

Our lab reported that *M. tuberculosis* PPS mutants are hypersensitive to Cu (18). We reasoned this phenotype may primarily be due to the repression of the RicR regulon in these strains. RicR is not a PPS substrate; therefore, it is not the accumulation of RicR itself that leads to regulon repression (15). Given that aldehyde accumulation in a PPS mutant sensitizes *M. tuberculosis* to NO, we hypothesized that aldehydes also cause Cu sensitivity. In this work, we determined that a mutation that prevents the accumulation of *p*HBA suppresses the Cu-sensitive phenotype of an *M. tuberculosis* PPS mutant. Additionally, we found *p*HBA can disrupt the production and activities of Cu-binding proteins. Finally, we showed that the aldehyde methylglyoxal (MG), which accumulates in macrophages during *M. tuberculosis* infections, can also reduce *M. tuberculosis* resistance to Cu.

## RESULTS

### Cu sensitivity of a *M. tuberculosis mpa* mutant was suppressed by disruption of *log*

**Fig 1:**
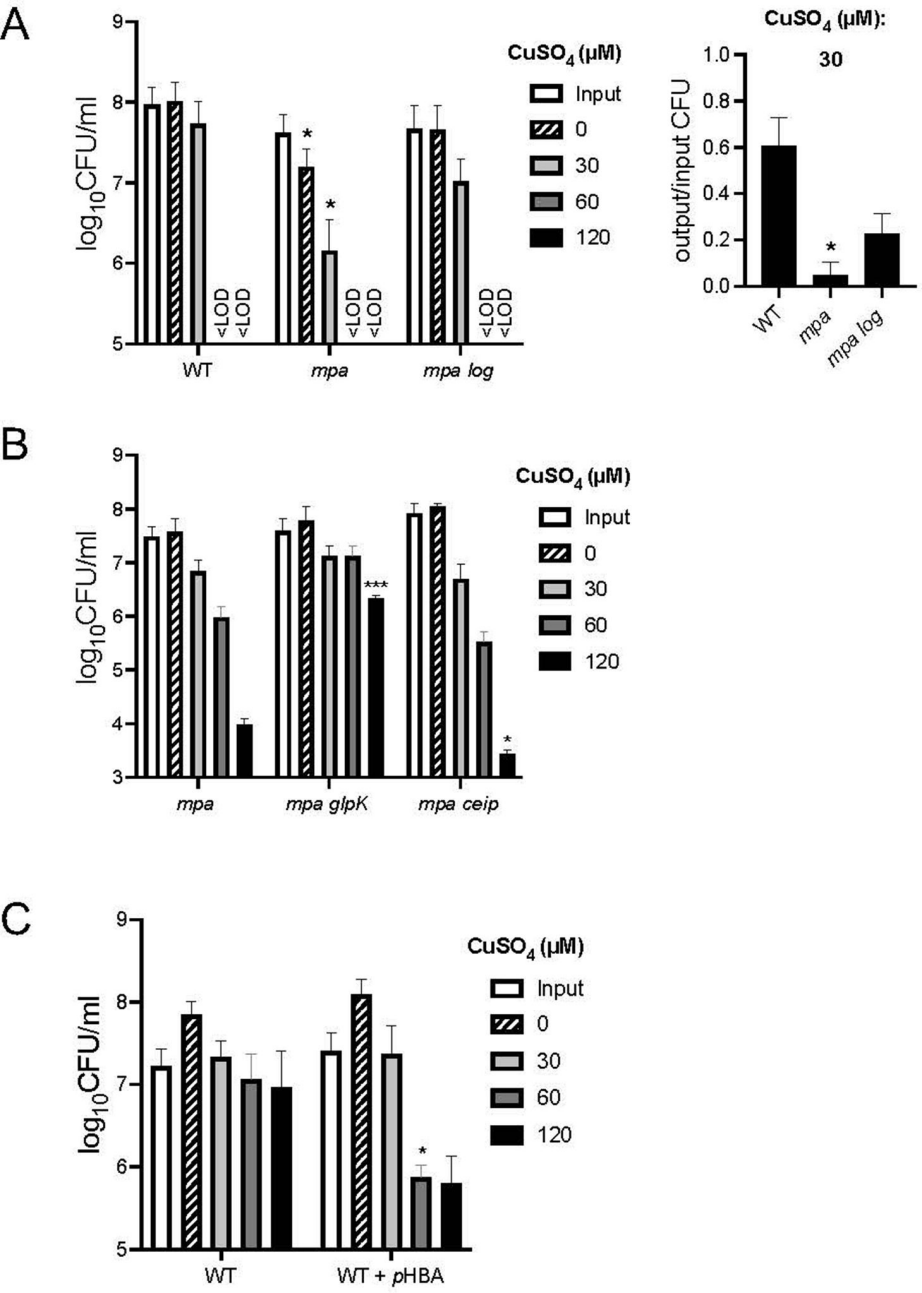
Copper (Cu) sensitivity of a Pup-proteasome system (PPS) mutant is suppressed by mutations in *log* or *glpK*. (**A**) Cu sensitivity assay shows a PPS *M. tuberculosis* mutant is hypersensitive to Cu. Bacteria were incubated for 10 days in the indicated CuSO_4_ concentrations. “Input” (white bars) indicates CFU at the beginning of the experiment, and “0” indicates how many bacteria were present after 10 days of incubation without added CuSO_4_ (striped bars). Bars represent mean with SD. “<LOD” indicates below the limit of detection (100 CFU). Significant differences were calculated, comparing CFU of strains to the first strain on the x-axis at the same CuSO_4_ concentration using an unpaired *t*-test with *P < 0.05; ***P < 0.001. Unlabeled bars showed no significant differences. Right panel: Given the slower growth of the *mpa* mutant, we calculated the ratio of the mean output CFU to mean input CFU for 30 µM CuSO_4_, which more accurately describes the survival of this strain in Cu. (**B**) Cu sensitivity assay performed as in (**A**) with previously characterized *mpa* strains suppressed for nitric oxide sensitivity. (**C**) Exogenous addition of *para*-hydroxybenzaldehyde (*p*HBA) sensitized wild-type (WT) *M. tuberculosis* to Cu. Cu sensitivity assays performed with WT *M. tuberculosis* incubated with or without 1.2 mM *p*HBA for 24 hours prior to addition of CuSO_4_. For all data shown, experiments are representative of at least two independent experiments, each done in technical triplicate.

*M. tuberculosis* strains with disruptions in *mpa* or *pafA* (PPS degradation-deficient strains) are hypersensitive to Cu (18) and NO (3). We previously showed that NO hypersensitivity is due to an accumulation of the PPS substrate Log; a transposon insertion in *log* in an *mpa* mutant suppresses its NO-sensitive phenotype (5). We tested if the disruption of *log* could also suppress the Cu-sensitive phenotype of an *mpa* mutant. We confirmed that a Δ*mpa::hyg* mutant, hereafter referred to as the *mpa* mutant, is sensitive to Cu (Fig. 1A, left panel, center bars; see Table 1 for all strains used in this work). Disruption of *log* (*log*::MycoMarT7) in the *mpa* mutant strain (hereafter referred to as an *mpa log* mutant) restored WT Cu resistance (Fig. 1A, left panel, right-side bars). Given that disruption of *log* prevents *p*HBA accumulation in the *mpa* mutant (5), this result suggested accumulation of *p*HBA in PPS mutants contributes to the Cu sensitivity of these strains.

**TABLE 1:** Strains, plasmids, and primers used in this study

Strain, plasmid, or primer	Phenotype, genotype, or sequence	Source or reference
***M. tuberculosis***		
H37Rv	WT, parental	ATCC^*a*^ 25618
MHD149	Hyg^R^; Δ*mpa*::*hyg*	(5)
MHD718	Hyg^R^ Kan^R^; Δ*mpa*::*hyg log*::MycoMarT7	(5)
MHD701	Hyg^R^; Δ*mymT*::*hyg*	(19)
MHD720	Hyg^R^ Kan^R^; Δ*mpa*::*hyg glpK*::MycoMarT7	(5)
MHD723	Hyg^R^ Kan^R^; Δ*mpa*::*hyg ceip*::MycoMarT7	(5)
MHD799	Hyg^R^; Δ*ricR*::*hyg*	This work
***E. coli***		
DH5α	F− ɸ80d*lacZ*ΔM15 Δ(*lacZYA-argF*)U169 *deoR recA1 endA1 hsdR17* (rΚ^−^mΚ^+^) *phoA supE44* λ-*thi1 gyrA96 relA1*	Gibco, BRL
**Plasmids**		
pYUB854	Hyg^R^; allelic exchange vector	(28)
pYUB854-*ricR*	Hyg^R^, *M. tuberculosis* Δ*ricR*::*hyg*	This work
**Primers**		
ricR-KpnI-F1	CAGGTACCATCGACGTCGGCGCGACTCATCAAAC	
ricR-XbaI-R1	GTTCTAGATCATTTACTCGCCATCTCCAGAGATAC	
ricR-H3-F2	CAAAGCTTTGATCGCCGCGTGTTGAAGCGCAAAC	
ricR-Spe1-R2	GTACTAGTTGACCTTGACCAGGCGAGGATTACG	

^*a*^ American Type Culture Collection.

Previous work by our lab found two additional mutations that suppress the NO sensitivity of an *mpa* mutant (5). These suppressor mutations are in *glpK*, which encodes a probable glycerol kinase, and in the promoter of *cei*, which encodes a possible secreted alanine-rich protein involved in cell membrane integrity (21). We found the *mpa glpK* (Δ*mpa::hyg glpK::*MycoMarT7) double-mutant had increased Cu resistance compared to the parental *mpa* strain (Fig. 1B, center bars). GlpK phosphorylates glycerol, resulting in the production of several aldehydes including MG and glyceraldehyde-3-phosphate. Thus, disruption of *glpK* might reduce the overall aldehyde burden in the *mpa* mutant, mitigating the negative effects of *p*HBA accumulation in this strain. In contrast, the *mpa ceip* (Δ*mpa::hyg ceip::*MycoMarT7) mutant was more sensitive to Cu than the *mpa* mutant (Fig. 1B, right side bars). Given that defects in *cei* increase membrane permeability (21), it is possible its reduced expression makes the bacterial cytosol more accessible to Cu.

We next tested if exogenously added *p*HBA could sensitize WT *M. tuberculosis* to Cu. We used 1.2 mM *p*HBA for all experiments in this study because this concentration, which is non-toxic on its own, can robustly synergize with NO to sterilize *M. tuberculosis* cultures (5). We preincubated WT *M. tuberculosis* with *p*HBA in minimal media for 24 hours before exposing the bacteria to Cu, reasoning that pre-treatment with *p*HBA would allow for transcriptional or other changes that affect Cu resistance. Additionally, *p*HBA added concomitantly with CuSO_4_ appeared to inhibit Cu-dependent killing for unclear reasons. As previously reported, *p*HBA alone had no effect on CFUs recovered (Fig. 1C, striped bars) (5). Pre-incubation of bacteria with *p*HBA reduced CFU after Cu treatment compared to Cu treatment alone (Fig. 1C, compare dark gray and black bars between untreated and *p*HBA-treated). Thus, endogenously produced or exogenously added *p*HBA sensitized *M. tuberculosis* to Cu.

### Expression of Cu-responsive genes was reduced in *p*HBA-treated *M. tuberculosis*

**Fig 2:**
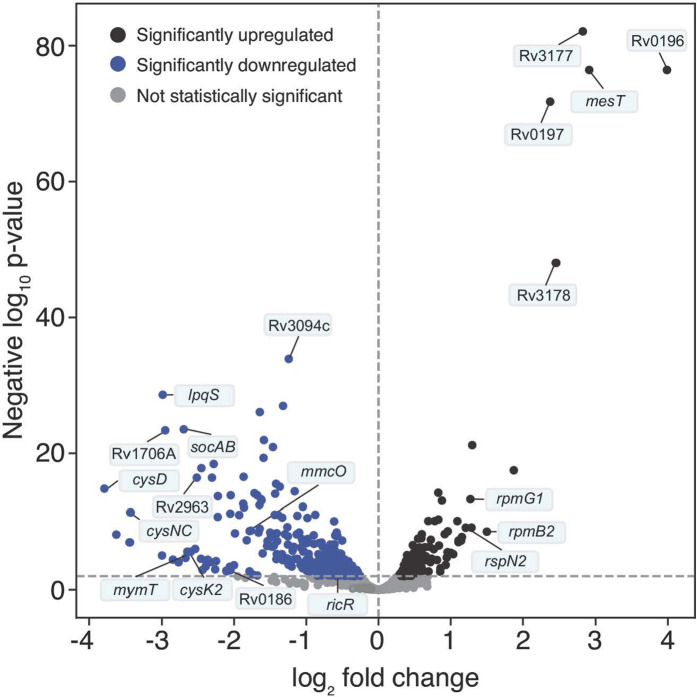
Volcano plot of significantly regulated genes in untreated versus *para*-hydroxybenzaldehyde-treated *M. tuberculosis*. x-axis: log_2_ fold change; y-axis: negative log_10_ *P*-value. Dotted horizontal line indicates cutoff for genes to be considered significantly differentially regulated (*P* < 0.01). Dotted vertical line indicates division between upregulated (black dots) and downregulated (blue dots) genes (log_2_ fold change = 0). Among the labeled genes are the RicR regulon (Rv0846, *mmcO*, *lpqS*, Rv1706A, *socAB*, *mymT*, *cysK2*, Rv2963, *ricR*) and select members of the Zur regulon (*rpmB2, rpsN2*, *rpmG1*).

Microarray analysis of PPS mutants lacking either *mpa* or *pafA* resulted in the identification of five promoters controlled by the DNA binding protein RicR (15). RicR dissociates from DNA in the presence of Cu, leading to the expression of genes required for robust Cu resistance and virulence (15, 18). Another Cu responsive operon, the CsoR operon, is also repressed in PPS mutant strains. Like RicR, CsoR releases repression of its operon after binding to Cu (13, 22). The CsoR operon includes *ctpV*, which encodes a cation transporter that contributes to Cu resistance and virulence (17). Given that PPS mutants are Cu sensitive and have reduced expression of two Cu responsive systems, we hypothesized *p*HBA accumulation in PPS mutants was responsible for the gene expression changes. To test this hypothesis, we performed RNA-Seq on WT *M. tuberculosis* treated with *p*HBA (Fig. 2; Table S1). We found that of 3,979 genes analyzed, six genes were significantly differentially upregulated more than twofold, and 35 genes were significantly differentially downregulated by more than twofold. Functional gene set enrichment analysis revealed significant downregulation of processes related to diverse metal ions, including Cu and iron, oxidative stress, and sulfur metabolism (Table S2). Remarkably, there was considerable overlap in the gene expression profiles of PPS mutants versus WT *M. tuberculosis* (15) and *p*HBA-treated bacteria versus untreated WT bacteria (Table 2 and 3, gray rows), which is reflected in the large overlap of functional gene sets with significantly altered expression (Table S2). The substantial overlap in the transcriptomes supported our hypothesis that *p*HBA contributes to the transcriptional signature of PPS mutants. Importantly, all members of the Cu-sensing regulons were downregulated in PPS mutants and *p*HBA-treated *M. tuberculosis* (Fig. 2; Table S1).

**TABLE 2:** Downregulated operons and regulons in WT *M. tuberculosis* treated with *p*HBA relative to untreated bacteria^*a*^

Regulator or locus	Gene	Product name	Fold change	*P*-value
**RicR**				
	*bglS*	Probable beta-glucosidase	0.24	2.77E-05
	*mymT*	Mycobacterial metallothionein	0.16	7.54E-08
	*mmcO*	Mycobacterial multicopper oxidase	0.29	3.08E-11
	*lpqS*	Probable lipoprotein	0.13	4.87E-32
	*cysK2*	Putative cysteine synthase	0.17	7.93E-08
	Rv0849	Probable conserved integral membrane transport protein	0.19	2.67E-05
	Rv1706A	Conserved hypothetical protein	0.13	1.23E-26
	Rv2963	Probable integral membrane protein	0.17	2.10E-19
	*socAB*	Small ORF induced by copper A and B	0.15	7.99E-27
	*ricR*	Regulated in copper repressor	0.67	5.40E-04
**CsoR**				
	Rv0968	Conserved protein	0.43	2.63E-05
	*ctpV*	Probable metal cation transporter P-type ATPase	0.37	1.63E-02
	Rv0970	Probable conserved integral membrane protein	0.51	4.18E-06
	*csoR*	Copper-sensitive operon repressor	0.30	6.30E-04
***cysDNC***				
	*cysD*	Probable sulfate adenlyltransferase subunit 2	0.07	9.58E-18
	*cysNC*	Probable bifunctional enzyme sulfate adenlyltransferase subunit 1 and adenlylsulfate kinase	0.09	4.67E-14
	Rv1287	Conserved hypothetical protein	0.21	1.57E-21
	Rv1288	Conserved protein	0.36	3.57E-10
	Rv1289	Unknown protein	0.88	1.29E-01
**Rv0762c-Rv0771 (putative regulator Rv0576, probable ArsR family metalloregulatory protein**)				
	Rv0762c	Conserved hypothetical protein	0.24	7.09E-14
	Rv0763c	Possible ferodoxin	0.20	4.40E-06
	*cyp51*	Cytochrome P450 51	0.19	2.83E-06
	Rv0765c	Probable oxidoreductase	0.16	8.26E-07
	*cyp123*	Probable cytochrome P450 123	0.21	3.01E-06
	Rv0767c	Conserved hypothetical protein	0.18	2.82E-08
	*aldA*	Probable NAD dependent aldehyde dehydrogenase	0.14	1.66E-06
	Rv0769	Probable dehydrogenase/reductase	0.21	8.10E-05
	Rv0770	Probable dehydrogenase/reductase	0.18	1.01E-04
	Rv0771	Possible 4-carboxymuconolactone decarboxylase	0.25	9.04E-11
**Rv3249c-Rv3252c (putative regulator WhiB4**)				
	Rv3249c	Possible transcriptional regulatory protein (TetR family)	0.31	9.30E-04
	*rubB*	Probable rubredoxin B	0.23	1.79E-04
	*rubA*	Probable rubredoxin A	0.13	3.37E-07
	*alkB*	Probable transmembrane alkane 1-monooxygenase	0.15	4.72E-06
**Rv2912c-Rv2913c**				
	Rv2912c	Probable transcriptional regulatory protein (TetR family)	0.09	2.56E-09
	Rv2913c	Possible D-amino acid aminohydrolase (D-amino acid hydrolase)	0.08	1.46E-10
**Rv0790-Rv0791c**				
	Rv0790c	Hypothetical protein	0.18	1.13E-06
	Rv0791c	Conserved protein	0.19	1.72E-06
	Rv0792c	Probable transcriptional regulatory protein (GntR family)	0.20	2.07E-19
**Rv2620c-Rv2622**				
	Rv2620c	Probable conserved transmembrane protein	0.24	1.18E-04
	Rv2621c	Possible transcriptional regulatory protein	0.23	1.39E-04
	Rv2622	Possible methylase	0.21	2.46E-13
	*lipX*	PE family protein possible lipase	0.18	6.78E-21
	Rv1586c	Probable PhiRv1 integrase	0.21	1.54E-16

^*a*^ Results are grouped by locus name or transcriptional regulator. All rows except *csoR* were more than twofold downregulated in PPS degradation-deficient strains (15). *p*HBA, *para*-hydroxybenzaldehyde; WT, wild-type.

**TABLE 3:** Upregulated operons and regulons in WT *M. tuberculosis* treated with *p*HBA relative to untreated bacteria^*a*^

Regulator or locus	Gene	Product name	Fold change	*P*-value
**Rv3173c-Rv3178**				
	Rv3173c	Probable transcriptional regulatory protein (probable TetR/AcR family)	3.66	1.48E-20
	Rv3174	Probable short-chain dehydrogenase or reductase	27.58	0
	Rv3175	Possible amidase	2.31	1.11E-11
	*mesT*	Probable epoxide hydrolase	7.52	3.85E-80
	Rv3177	Possible peroxidase	7.07	4.08E-86
	Rv3178	Conserved hypothetical protein	5.48	1.41E-51
**Rv0195-Rv0197**				
	Rv0195	Possible two-component transcriptional regulatory protein (LuxR family)	2.46	2.17E-24
	Rv0196	Possible transcriptional regulatory protein	15.86	2.99E-80
	Rv0197	Possible oxidoreductase	5.19	2.21E-75
**Zur**				
	Rv0106	Conserved hypothetical protein	2.15	2.32E-09
	Rv0280	PPE family protein PPE3	1.81	5.17E-05
	Rv0281	Possible S-adenoslymethionine-dependent methyltransferase	1.53	1.38E-04
	*eccA3*	ESX-3 conserved component type VII secretion system (T7SS) protein	1.41	2.99E-05
	*eccB3*	ESX-3 T7SS protein	1.42	1.06E-04
	*eccC3*	ESX-3 T7SS protein	1.41	2.94E-05
	Rv0286	PPE family protein PPE4	1.19	1.04E-01
	*esxG*	ESAT-6 like protein	1.27	1.69E-02
	*esxH*	Low molecular weight protein antigen 7	1.29	9.20E-03
	*espG3*	ESX-3 secretion-associated protein	1.19	5.15E-02
	*eccD3*	ESX-3 T7SS probable transmembrane protein	1.15	1.99E-01
	*mycP3*	Probable membrane-anchored mycosin	1.21	1.89E-02
	*eccE3*	ESX-3 T7SS secretion system protein	1.14	1.03E-01
	*rpsR2*	30S ribosomal protein S18	2.00	1.18E-07
	*rpsN2*	30S ribosomal protein S14	2.44	1.06E-11
	*rpmG1*	50S ribosomal protein L33	2.42	4.54E-16
	*rpmb2*	50S ribosomal protein L28	2.83	4.58E-11
	Rv2059	Conserved hypothetical protein	1.84	7.93E-16
	*zur*	Zinc uptake regulator protein	0.92	2.23E-01

^*a*^ Results are grouped by locus name or transcriptional regulator. All Zur rows except *zur* were more than twofold upregulated in PPS degradation-deficient strains (15). *p*HBA, *para*-hydroxybenzaldehyde; WT, wild-type.

**Fig 3:**
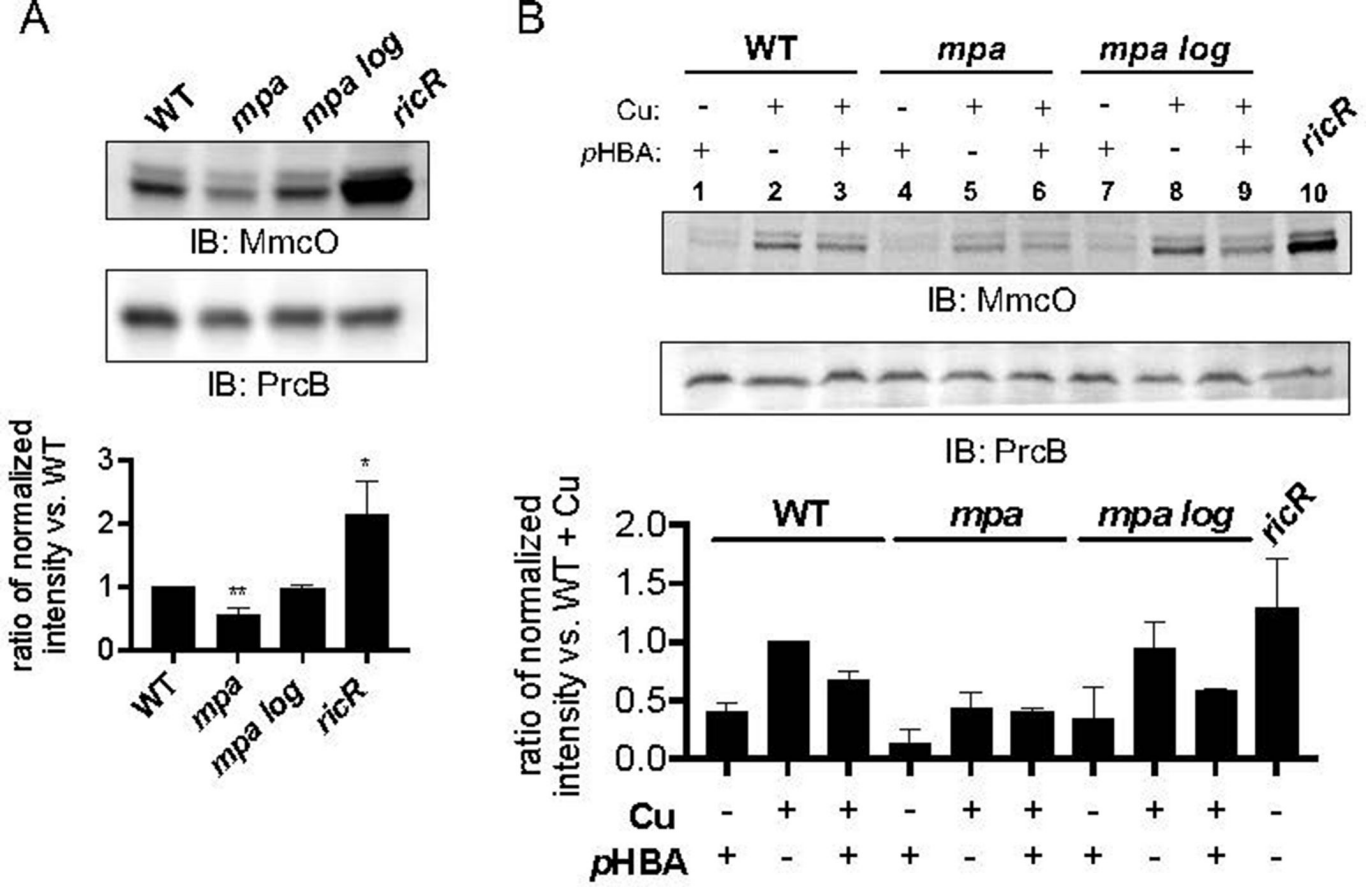
MmcO levels are reduced in a Pup-proteasome system mutant and in *para*-hydroxybenzaldehyde (*p*HBA)-treated *M. tuberculosis*. (**A**) *M. tuberculosis* strains indicated were grown in Sauton minimal media and treated with 50 µM CuSO_4_ for 24 hours prior to collection of equivalent amounts of bacteria for whole-cell lysates and preparation for immunoblotting. Proteins were detected using polyclonal rabbit antibodies raised against MmcO or the β-subunit of the proteasome (PrcB) as a loading control. This experiment is a representative of three biological replicates. Below: quantification of band intensity from three independent experiments with MmcO levels normalized to PrcB levels before calculating the intensity ratio between each strain compared to the wild-type (WT) strain. Bars indicate mean with SD error bars. Significance was calculated using an unpaired *t*-test to WT with *P < 0.05; **P < 0.01. Unlabeled bars showed no significant differences. (**B**) Immunoblotting for MmcO in strains treated with or without 1.2 mM *p*HBA for 4 hours before treatment with CuSO_4_. Below: quantification as in (**A**) of MmcO levels normalized to PrcB levels for each strain and condition, relative to MmcO levels in the WT strain treated with copper (Cu) only. Data are representative of two independent experiments. Bars indicate mean with SD error bars.

While the RicR regulon encodes eight genes, only two (excluding *ricR*) have been implicated in Cu resistance: *mmcO* and *mymT*, with a *mymT* mutant having the strongest Cu-sensitive phenotype (18, 19). To follow up the transcriptional analysis, we sought to determine if protein levels of either of these key Cu resistance proteins were reduced in *p*HBA-treated bacteria or a PPS mutant. Given the low abundance and small size (53 amino acids) of MymT in WT *M. tuberculosis* (15, 19), we instead looked at MmcO. MmcO is a membrane-associated Cu oxidase that is hypothesized to convert Cu(I) into less toxic Cu(II) based on its high similarity to other Cu oxidases (16, 18). We assessed levels of MmcO in Cu-treated *mpa, mpa log,* and *ricR* null (Δ*ricR::hyg*) strains relative to a WT parental strain. As previously reported, the *ricR* null mutant over-produces MmcO relative to WT bacteria; in contrast, the *mpa* mutant had approximately two-fold less MmcO relative to WT bacteria (Fig. 3A). Consistent with the suppressive effect of the *log* mutation on Cu sensitivity in a PPS mutant (Fig. 1A), the *mpa log* strain had WT levels of MmcO (Fig. 3A).

We next determined if exogenously added *p*HBA could affect MmcO levels. In the absence of Cu, *p*HBA treatment alone resulted in low MmcO levels in all strains (Fig. 3B, lanes 1, 4, and 7) (16, 18). Cu treatment of the WT strain increased MmcO levels as previously reported (16, 18) (Fig. 3B, lanes 1 v. 2), whereas *p*HBA reduced levels of MmcO produced in Cu-treated WT bacteria by approximately one-third (Fig. 3B, lanes 2 v. 3). Even with the addition of Cu, the *mpa* strain showed low levels of MmcO, nearly half the level observed in Cu-treated WT bacteria (Fig. 3B, lanes 2 v. 5), and the addition of *p*HBA had a minor effect on the already low MmcO levels in the *mpa* mutant (Fig. 3B, lanes 5 v. 6). MmcO levels in the *mpa log* strain were similar to that of the WT strain (Fig. 3B, lanes 2v. 8, and lanes 3 v. 9). Together, these results indicate that the presence of endogenously produced or exogenously added *p*HBA reduced the levels of at least one RicR-regulated gene product, which supports our transcriptional data.

### *p*HBA altered the function of the major Cu resistance protein, MymT

**Fig 4:**
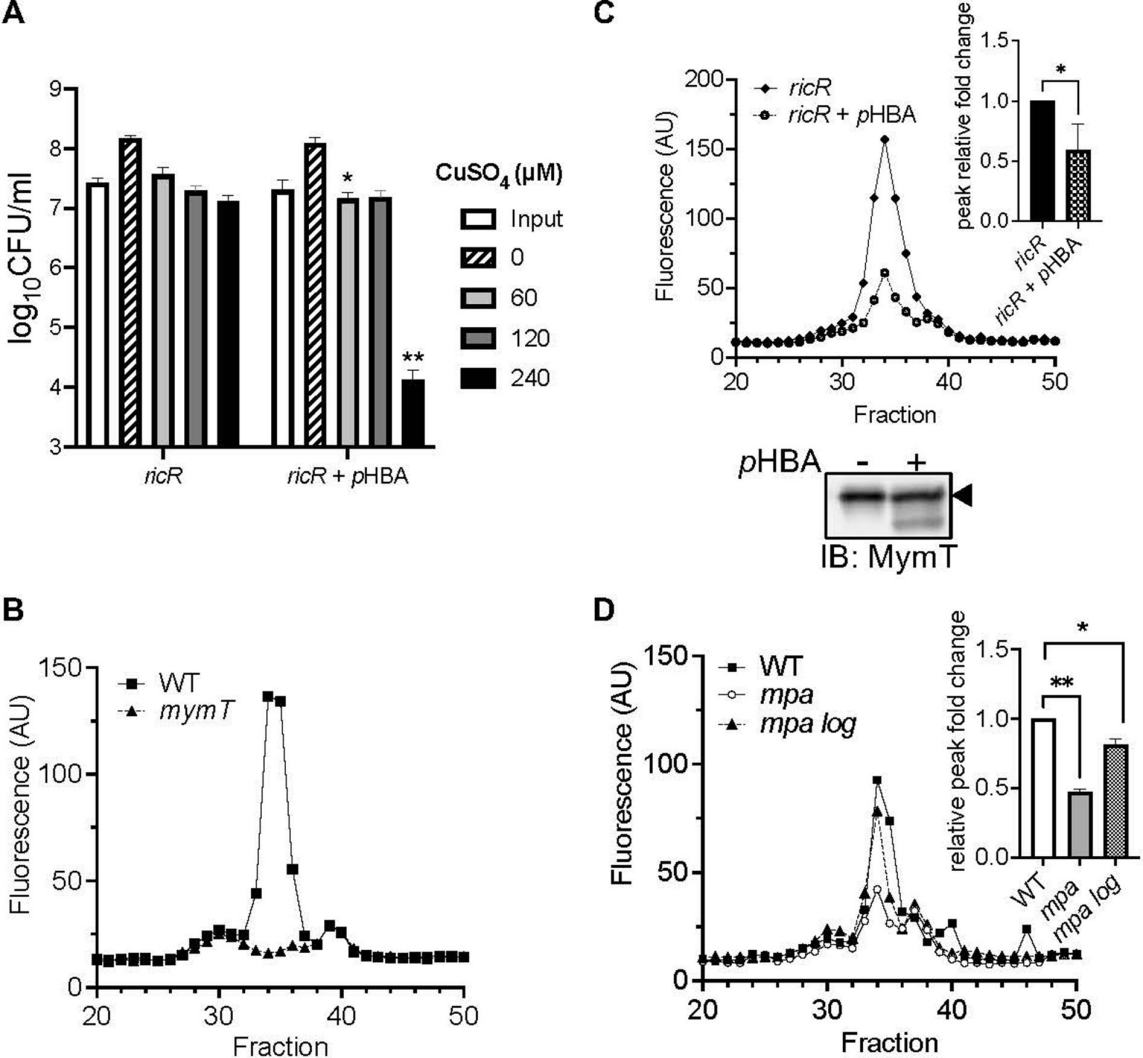
MymT activity is reduced in the presence of *p*HBA. (**A**) Cu sensitivity assay of a *ricR* mutant after preincubation with *para*-hydroxybenzaldehyde (*p*HBA). Data are representative of two independent experiments, each done in technical triplicate. (**B**) MymT Cu(I)-thiolate luminescence (excitation = 280 nm, emission = 595 nm, cutoff = 325 nm). Wild-type (WT) *M. tuberculosis* lysates exhibited a peak in luminescence in fraction 34, which was abolished in lysates of a *mymT* mutant. (**C**) Fractionated lysates from *M. tuberculosis ricR* mutant strain treated with or without *p*HBA. Inset: quantification of fold change between two experiments. Lower section: immunoblot for MymT of pooled fractions corresponding to the maximal fluorescence peak. Arrowhead indicates full-length MymT. (**D**) Fractionated *M. tuberculosis* lysates from WT, *mpa,* and *mpa log* strains. Inset: quantification of fold change between two independent experiments. All quantifications were analyzed for significance using an unpaired *t*-test with *P < 0.05; **P < 0.01. Unlabeled bars did not show significant differences.

Up to this point, our data support a model whereby PPS-defective *M. tuberculosis* are hypersusceptible to Cu due to a reduction in Cu-responsive gene expression caused by *p*HBA. However, we could not rule out the possibility that *p*HBA directly disrupts the function of one or more of the Cu-responsive proteins. A way to test this hypothesis is to measure the effect *p*HBA has on a *ricR* null mutant, which constitutively expresses high levels of all of the RicR regulon genes. We performed a Cu sensitivity assay on a *ricR* mutant pretreated with *p*HBA. Because *ricR* mutants are highly resistant to Cu (15), we needed to use a higher Cu concentration than in previous experiments to kill the bacteria. At the highest CuSO_4_ concentration used, *p*HBA robustly sensitized the *ricR* mutant to Cu (Fig. 4A, black bars), suggesting that repression of the RicR regulon was not the only mechanism by which *p*HBA sensitized *M. tuberculosis* to Cu.

Given that the metallothionein MymT plays a dominant role in Cu resistance in *M. tuberculosis*, we hypothesized that *p*HBA could affect the function of MymT. Metallothioneins are found in all domains of life and protect cells from the potentially harmful effects of free Cu (23). MymT uses cysteines to bind reduced Cu [Cu(I)] (19). Given that aldehydes can readily form hemiacetals with cysteines and disable protein function (24), we hypothesized that *p*HBA reacts with cysteines in MymT, preventing its ability to bind and sequester toxic Cu. Cu metallothioneins form solvent-shielded Cu(I)-thiolate cores that luminesce when excited by UV light (19, 25). Thus, a reduction in luminescence would indicate decreased Cu binding.

We first measured MymT Cu-thiolate cores from WT *M. tuberculosis* by fractionating whole-cell lysates of Cu-treated bacteria and measuring luminescence emitted from each fraction after excitation by UV light. A sharp peak of luminescence was observed in the fractionation profile that could be attributed to MymT, given that this peak was absent from lysates of a *mymT* null mutant (Table 1 Fig. 4B). We next examined the effect of *p*HBA on MymT luminescence in the *ricR* mutant. We observed a reduction of the MymT luminescence peak in lysates collected from the *ricR* mutant treated with *p*HBA compared to that of untreated bacteria, strongly suggesting *p*HBA affected the ability of MymT to bind Cu (Fig. 4C). To ensure equivalent MymT levels in *ricR* mutant lysates treated with or without *p*HBA, we pooled and concentrated the three fractions corresponding to the maximal MymT luminescence peak for immunoblot analysis. We found MymT levels were high and equivalent in *ricR* mutant fractions, whether or not they were *p*HBA-treated (Fig. 4C, lower panel). Interestingly, we consistently detected a smaller species of MymT in the *p*HBA-treated samples. It is possible this smaller species was caused by a direct modification of MymT by *p*HBA, an idea that remains to be tested.

We also tested if MymT luminescence was reduced in a *M. tuberculosis* PPS mutant compared to in the parental strain. The MymT luminescence peak in the *mpa* strain was significantly lower, and this reduction was restored to near WT levels in the *mpa log* double mutant (Fig. 4D). These data support the hypothesis that the accumulation of *p*HBA reduced either the amount or activity, or both, of MymT in a PPS mutant.

### A metabolic aldehyde sensitized *M. tuberculosis* to Cu

**Fig 5:**
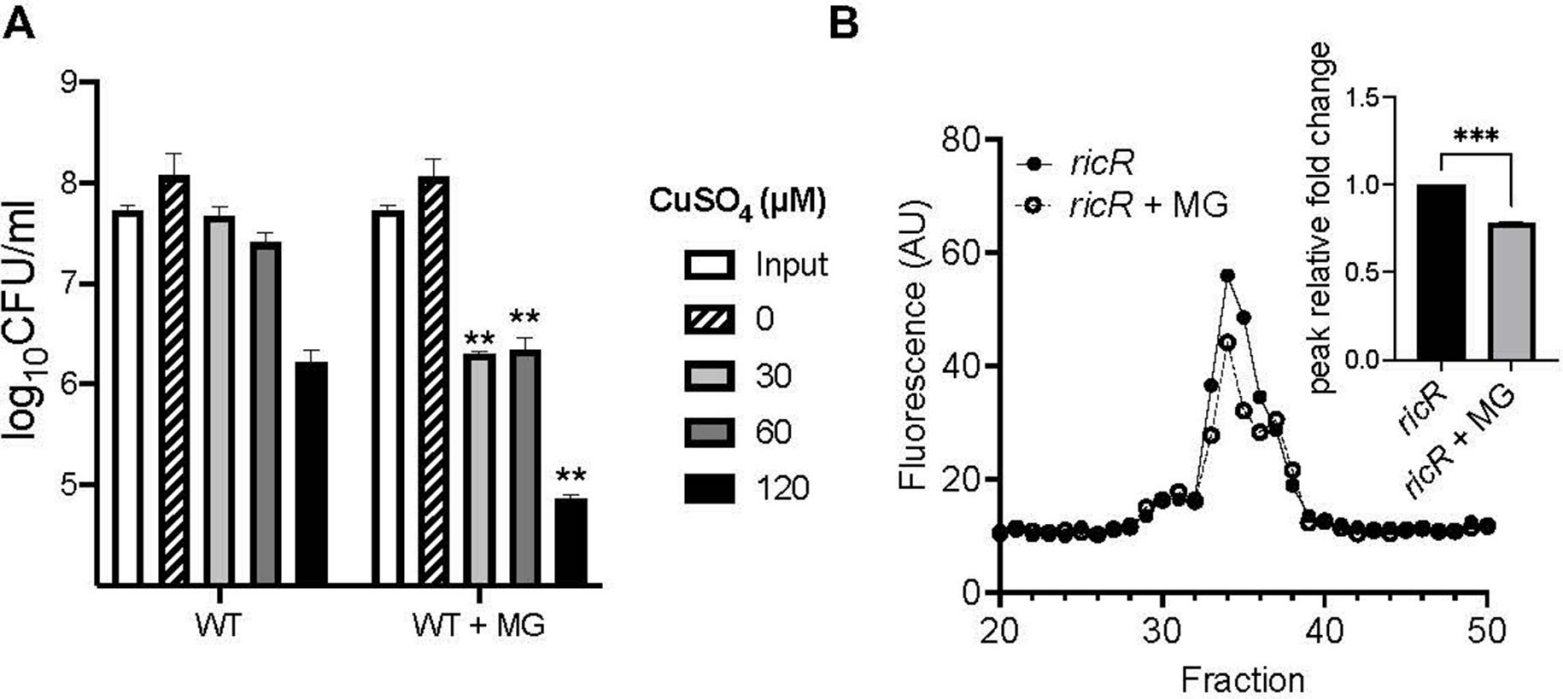
Methylglyoxal sensitizes *M. tuberculosis* to copper (Cu) and disrupts MymT Cu binding. (**A**) Cu sensitivity assay with the addition of methylglyoxal (MG) to wild-type (WT) bacteria at day 0. Significant difference was calculated comparing with the first strain on the x-axis at the same CuSO_4_ concentration using an unpaired *t*-test with ***P < 0.001. (**B**) MymT Cu(I)-thiolate luminescence with an *M. tuberculosis ricR* mutant treated with or without MG. Inset: quantification of peak fold change relative to untreated, analyzed for significance using a two-tailed *t*-test (*P* < 0.05). Data are representative of two experiments.

A recent hypothesis put forward by the Darwin and Stanley labs proposes host cell-derived aldehydes of metabolism contribute to bacterial control during infections (26). Macrophages undergo an increase in aerobic glycolysis, known as the Warburg Effect, following infection with *M. tuberculosis* and other pathogens (27 - 30). A by-product of aerobic glycolysis is MG, also known as pyruvaldehyde, which has been detected at millimolar concentrations in *M. tuberculosis*-infected mouse macrophages (31). Unlike with *p*HBA, MG pretreatment resulted in increased Cu resistance, leading us to hypothesize that MG preincubation specifically induced either an aldehyde or Cu resistance pathway, an idea we are testing. Nonetheless, when simultaneously added to bacteria with Cu, MG at normally non-toxic levels synergized with Cu to robustly kill *M. tuberculosis* (Fig. 5A, right panel, gray and black bars). Furthermore, MG treatment reduced luminescence of MymT in a *ricR* mutant (Fig. 5B). Overall, this result indicates that a physiologic aldehyde that is present during infections has the potential to sensitize *M. tuberculosis* to Cu by disrupting one or more Cu-responsive proteins.

## DISCUSSION

**Fig 6:**
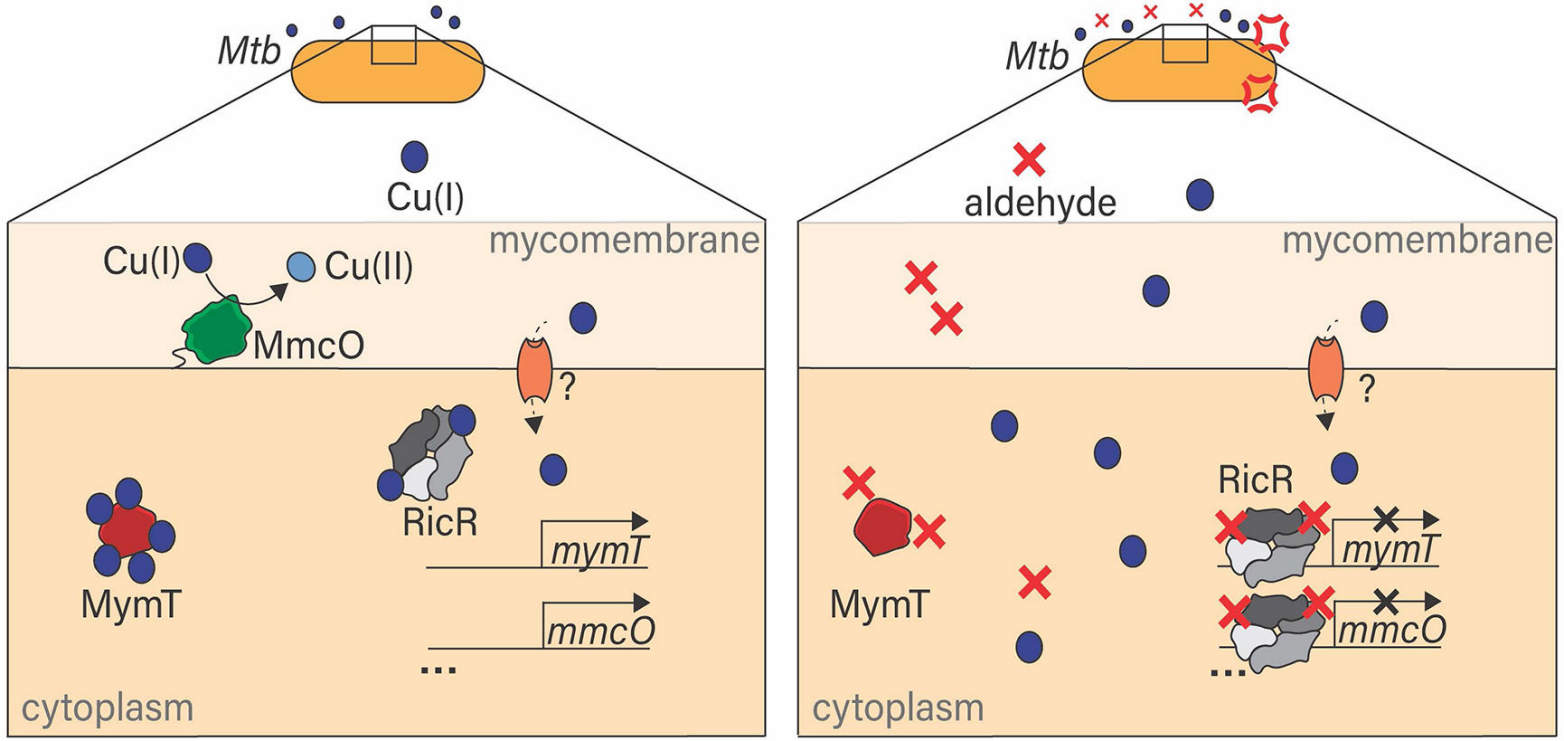
Proposed model of aldehyde sensitization of *M. tuberculosis* to copper (Cu). Left: in the absence of aldehyde, Cu(I) enters the cytoplasmic space through unknown transporters. Cu prevents RicR from binding to DNA, allowing expression of the RicR regulon genes, some of which mitigate Cu(I) toxicity, MymT sequesters Cu(I) to prevent it from damaging the cell, and MmcO is a periplasmic multicopper oxidase that oxidizes Cu(I) to less toxic Cu(II). Right: in the presence of aldehyde, RicR cannot release from DNA, even in the presence of Cu, leading to repression of the RicR regulon and the observed Cu sensitivity of aldehyde-treated and Pup-proteasome system mutant *M. tuberculosis* strains. Additionally, MymT is unable to bind Cu in the presence of aldehyde.

In this work, we sought to test if the Cu-sensitive phenotype of an *M. tuberculosis* PPS mutant was due to an accumulation of the aldehyde *p*HBA. We found that elimination of the PPS substrate Log, which is the source of *p*HBA, restored Cu resistance to WT levels. Furthermore, addition of *p*HBA to WT *M. tuberculosis* cultures was sufficient to sensitize bacteria to Cu. Cu sensitization in both PPS mutants and *p*HBA-treated WT *M. tuberculosis* was likely due to the reduced expression of genes needed for Cu resistance. We also showed that the Cu-binding function of MymT was altered in the presence of *p*HBA, possibly by disrupting cysteines in the protein. Finally, we showed that MG, an aldehyde produced by activated macrophages, also sensitized *M. tuberculosis* to Cu *in vitro*. Collectively, we propose a model whereby *p*HBA and other aldehydes can directly or indirectly disable Cu sensing by RicR, leading to the constitutive repression of the RicR regulon, and also disrupt Cu binding by MymT, preventing its ability to confer Cu resistance (Fig. 6).

In this study we also showed that a transposon disruption in *glpK*, which encodes a glycerol kinase, suppressed the Cu sensitive phenotype of a PPS mutant. This mutation also suppresses NO sensitivity (5). Along these lines, several independent studies recently reported that *glpK* mutations are often found in clinical *M. tuberculosis* isolates (32 - 34). These mutations are reversible and confer decreased susceptibility to antituberculosis drugs, suggesting phase variation occurs in response to antibiotic pressure on the bacteria. The Alland group proposed that the activation of a general stress response following a block in glycerol metabolism provides increased antibiotic tolerance (32). Similar to our hypothesis that a *glpK* mutation reduces aldehyde levels in PPS mutants, the Sassetti lab proposed that a block in glycerol metabolism, which produces MG, could protect bacteria by lowering endogenous aldehyde burden; this idea suggests aldehydes could sensitize bacteria to antibiotics (33).

In addition to the RicR regulon, the metal-responsive CsoR operon and Zur regulon were similarly regulated between *p*HBA-treated WT bacteria and PPS mutants. CsoR represses the expression of *ctpV*, which is a cation transporter involved in Cu resistance (17) and is repressed in PPS mutants and WT bacteria incubated with *p*HBA. CsoR and RicR are paralogs that use cysteines to sense Cu (13, 18); thus, it is possible that *p*HBA also disrupts the ability of CsoR to bind Cu, resulting in the constitutive repressed production of CtpV.

The Zur regulon was upregulated in *p*HBA-treated *M. tuberculosis* (Table 3) and in PPS mutants (15). Unlike the Cu regulators, Zur is a zinc-responsive regulator that binds to and represses its promoters in the presence of excess zinc (35). Zur-regulated genes are thus induced in low zinc and implicated in zinc uptake (35). The ESAT-6 cluster 3 (ESX-3) genes in the Zur regulon are also regulated by iron-dependent repressor (IdeR) (36). However, no other IdeR-dependent genes were differentially expressed between untreated and *p*HBA-treated *M. tuberculosis*, similar to what we observed in PPS mutants (15). Notably, unlike the Cu regulators and Zur, IdeR does not use cysteine to coordinate iron.

In addition to the Zur regulon, several genes were induced in *p*HBA-treated *M. tuberculosis* and not in PPS mutants (Table 3). Some of these genes, e.g., *mesT* and Rv0195, are upregulated in hypoxia or after damage to the mycomembrane (37 - 39). It is possible that these genes are specifically induced in response to extracellular aldehyde exposure and not by endogenously produced aldehyde. Alternatively, the amount of *p*HBA we used for RNA-Seq cultures induced a transcriptional response that would not be achieved by what are likely much lower *p*HBA concentrations found in PPS mutants.

We also observed several uncharacterized operons with gene expression patterns shared between PPS mutants and *p*HBA-treated WT bacteria, including: the *cysDNC* operon, which encodes a sulfate-activating enzyme complex that is implicated in virulence, oxidative stress, sulfate limitation, and sensing of exogenous cysteine (40); Rv0762c-Rv0771, which includes a gene for a putative aldehyde dehydrogenase (*aldA*) and is predicted to be regulated by a probable ArsR-like metalloregulatory transcriptional repressor (Rv0576) (41, 42); and Rv3249c-Rv3252c that is predicted to be controlled by WhiB4, a redox-responsive, iron–sulfur cluster-containing transcriptional repressor (Table 2; Table S2) (43). Rv3249c-Rv3252c encodes the rubredoxins RubA and RubB and a putative monooxygenase AlkB. Relevantly, many of these genes are predicted to be regulated by metal-binding proteins that use cysteines to coordinate their respective metals (42, 43). Because aldehydes can form adducts with thiols in proteins, it is possible that cysteine-dependent metal coordinating proteins are particularly sensitive to aldehyde exposure.

Our data also suggest that aldehydes have a direct effect on the function of MymT. We observed that *p*HBA and MG reduced MymT luminescence, suggesting these aldehydes directly disrupted Cu binding to MymT. An alternative explanation is that *p*HBA quenched MymT luminescence rather than directly preventing MymT Cu binding; however, a smaller species of MymT formed only after *p*HBA treatment, suggesting this aldehyde directly affected the structure of MymT. This smaller species of MymT may have formed a more compact conformation due to a covalent interaction with *p*HBA, allowing it to migrate through SDS-PAGE gels more quickly. Alternatively, *p*HBA-modified MymT could have adopted a conformation that exposed it to peptidases, resulting in partially degraded MymT. Unlike MymT, we could not test whether or not *p*HBA directly affected MmcO activity given that MmcO is a membrane-anchored protein without an established activity assay. Although MmcO is not predicted to use cysteines to function, its activity may nonetheless be affected by aldehydes, a hypothesis that remains to be tested.

A recent study by the Glickman lab identified an integrated system involving Rip1 protease and the PdtaS/R two-component system in *M. tuberculosis* that senses and mediates resistance to Cu and NO (44). The NO sensitivity of a *rip1* mutant is attributed to a block in chalkophore biosynthesis. Chalkophores bind to Cu with high affinity (44 - 46), and a follow-up study supports a model that mycobacteria require chalkophores to acquire Cu during low Cu conditions, i.e., this system is not for Cu resistance *per se* but for Cu acquisition (47). While it remains to be determined how Cu and NO resistance is conferred by Rip1, it is unlikely that aldehydes are involved given that known Cu resistance genes are not repressed in a *rip1* mutant. Importantly, these data suggest there are additional ways for *M. tuberculosis* to be sensitized to NO and Cu.

An active anti-microbial role for aldehydes *in vivo* has yet to be established. In macrophages, aldehydes are produced at low levels during cellular metabolism (48, 49), but a shift to aerobic glycolysis following infection with *M. tuberculosis* leads to an increase of aldehydes such as glyceraldehyde-3-phosphate and MG (27 - 31 - 50 - 52 - 52). Induction of aerobic glycolysis plays a role in infection control given that the inhibition of this pathway in mouse macrophages leads to loss of some interferon-γ-dependent control of *M. tuberculosis* growth (27). Thus, aldehydes produced during this shift to glycolysis might contribute to antibacterial activity for the host.

More broadly, aldehydes may also have a role in the defense against other microbes. For example, a recent report by the Portnoy lab showed that in *Listeria monocytogenes,* MG activates transcription of glutathione (GSH) synthase-encoding gene *gshF*, leading to increased GSH production and thereby activating the master virulence regulator PrfA (53). A *L. monocytogenes* mutant that lacks a gene-encoding glyoxalase A, a key enzyme in MG detoxification, has decreased GSH levels *in vitro* and is attenuated for infection. Together, these results suggest that MG is an important cue for some pathogens to turn on virulence programming.

Our data provide the first evidence that aldehydes can sensitize *M. tuberculosis* to Cu. Importantly, despite well-established evidence of their toxicity, the antibacterial mechanisms of aldehydes are relatively uncharacterized; thus, our study may begin to provide new insight into how aldehydes can target and inactivate bacterial pathways needed for survival.

## MATERIALS AND METHODS

### Bacterial strains, growth conditions, plasmids, and primers

The bacterial strains, plasmids, and primers used in this work are listed in Table 1. *Mycobacterium tuberculosis* strains were grown in 7H9 liquid media (Difco) supplemented with 0.2% glycerol, 0.05% Tween 80, 0.5% bovine serum albumin (BSA), 0.2% dextrose, and 0.085% sodium chloride (ADN) (referred to as “7H9c” from here on) or Sauton minimal media (3.7 mM potassium phosphate, monobasic; 2.4 mM magnesium sulfate; 30 mM L-asparagine; 3.5 mM zinc sulfate; 9.5 mM citric acid; 6.0% glycerol; 0.005% ferric ammonium citrate; 0.05% Tween-80). Cultures were grown at 37°C without agitation in vented flasks (Corning). For *M. tuberculosis* growth on solid media, Middlebrook 7H11 agar (Difco and Remel) was supplemented with Middlebrook OADC (oleic acid, albumin, dextrose, and catalase; BBL). *Mycobacterium tuberculosis* strains were grown in 50 µg/mL kanamycin, or 50 µg/mL hygromycin when necessary.

For CuSO_4_ solutions, stock solutions were made by dissolving the appropriate amount of CuSO_4_ powder (Fisher Scientific) in water and filter-sterilizing with a 0.45-µm filter. For aldehyde solutions, 50 mM *p*HBA was made with *p*HBA powder dissolved in water and filter-sterilized using a 0.45-µm filter. MG (5.5 M) was diluted in sterile water just before use. Both aldehydes were purchased from Sigma-Aldrich, Inc.

### RNA-Seq

*M. tuberculosis* cultures were grown in 7H9c media and treated with 1.2 mM *p*HBA at an optical density at 580 nm (OD_580_) of 1.0 (late logarithmic to early stationary phase) or left untreated. Twenty-four hours later, RNA was purified as previously described (15). RNA was isolated from three biological replicate cultures. Library preparation and Illumina HiSeq Sequencing were performed by GENEWIZ, LLC. Sequence reads were mapped to the *M. tuberculosis* H37Rv genome sequence (RefSeq identifier GCF_000195955.2) using bwa v0.7.17 (54) and sorted using samtools v1.9 (55). An average of 97.8% of reads were mapped to the reference genome, indicating high quality of samples. Given a locus of interest, *socAB*, was not included in the assembly annotation from RefSeq, we added it to the annotation files at genomic coordinates NC_000962.3:1,933,937-1,934,497. Using the alignment files generated for each sample, the *featureCounts* command in Subread v2.0.1 (56) was used to count the reads mapping to each gene in the reference. Read counts per gene and sample (feature counts) were loaded into R v4.2.0 (57) for further analysis using the package DESeq2 v1.36.0 (58). DESeq2’s normalization function was used to normalize the counts to make expression levels more comparable between the different samples. To compare gene expression changes between *p*HBA-treated and untreated samples, the Wald test was used to generate *P*-values and log_2_ fold changes. Genes with an adjusted *P*-value of <0.05 and fold change of >2.0, when comparing *p*HBA-treated to untreated *M. tuberculosis,* were considered differentially expressed genes (Table S1). Raw sequencing data files are available in a PATRIC public workspace:

(https://www.bv-brc.org/workspace/ginalimon@bvbrc/Limon_Darwin_RNA-Seq). The volcano plot was generated using python v3.10.6 and package bioinfokit v2.1.10 (59).

### Gene set enrichment analysis

Gene Ontology (GO) annotations for assembly *M. tuberculosis* H37Rv (Genbank ID: AL123456.3) were obtained via the QuickGO REST API by querying all locus tags in the assembly’s annotation. Gene set enrichment analyses were carried out for each of the three GO with topGO v.2.44.0 using the *weight01* algorithm and the Fisher statistic, using a *P*-value significance threshold of 0.05. For genes whose expression was deemed significantly upregulated (21 genes) or downregulated (38 genes) in a previous publication, the gene universe consisted of all genes targeted by the probes of the deployed microarray (GEO ID GPL4292). For genes whose expression was deemed significantly altered in this publication, the gene universe consisted of all genes annotated in assembly *M. tuberculosis* H37Rv (Genbank ID: AL123456.3).

### Cu sensitivity assay

Cu sensitivity assays were performed as previously described (15, 18). Briefly, *M. tuberculosis* strains were grown in 7H9c to an OD_580_ = 0.5–1.0. Bacteria were washed once with Sauton minimal media with no added Cu and collected using a low-speed centrifugation (150 *g*) to remove clumped cells. Supernatants containing mostly unclumped bacteria were diluted to OD_580_ = 0.08 in Sauton minimal media. One hundred ninety-four microliters of this diluted culture was transferred to 96-well plates; 6 µL of the appropriate stock concentration of CuSO_4_ or aldehyde or both was added to the desired final concentration. Plates were incubated at 37°C for 10 days; after which, cultures were diluted and inoculated onto 7H11 agar Y-plates. Plates were incubated for 14–21 days before enumerating CFU. As previously reported (15), we used a range of CuSO_4_ concentrations due to variability of Cu sensitivity between experiments. Each experiment was done at least twice in technical triplicate.

### *M. tuberculosis* lysate preparation for immunoblotting

For all blots, bacteria were grown in Sauton minimal media with no added Cu. For MmcO blots, at OD_580_ = 0.5–1.0, cultures were treated with 1.2 mM *p*HBA for 4 hours before treatment with 50 µM CuSO_4_. Bacteria were harvested 24 hours after the addition of CuSO_4_. Bacterial densities were measured and equivalent cell numbers were collected based on the OD_580_ of the cultures. For example, an “OD_580_ equivalent of 1” indicates the OD_580_ of a 1-mL culture is 1.0. For most assays, 5 OD_580_ units were collected and washed once with Dulbecco’s phosphate-buffered saline (Corning, Inc with 0.05% Tween-80) to remove BSA in 7H9c media. Bacterial pellets were then resuspended in 300 µL TE buffer (100 mM Tris-Cl, 1 mM EDTA pH 8.0), and transferred to bead-beating tubes with 200 µL zirconia beads; tubes were beaten for 30 seconds three times, with icing for 30 seconds in between in a mini bead beater (all materials from Bio-Spec). One hundred fifty microliters of lysate was transferred into new tubes with 50 µL 4✕ SDS sample buffer (250 mM Tris pH 6.8, 2% SDS, 20% β-mercaptoethanol, 40% glycerol, 1% bromophenol blue) and boiled at 100°C for 10 minutes. Proteins were separated by sodium dodecyl sulfate-polyacrylamide gel electrophoresis (SDS-PAGE) and transferred onto nitrocellulose membranes. Membranes were blocked in 3% milk or BSA prior to incubation with polyclonal rabbit antibodies as indicated in the figure legends. For loading controls, the same membranes were stripped with 0.2 N NaOH as described elsewhere (60) and re-blocked before incubation with antibodies to *M. smegmatis* PrcB (61).

### Construction of a Δ*ricR*::*hyg* mutant

We made a *M. tuberculosis ricR* deletion mutant strain using a previously described method (15). Briefly, pYUB854 (20) was used to clone sequences encompassing ~700 bp upstream (5′) and ~700 downstream (3′) of the *ricR* gene. The 5′ and 3′ sequences, including the start and stop codons, respectively, were cloned to flank the hygromycin resistance cassette in pYUB854. The plasmid was digested with PacI, and approximately 1 µg of linearized, gel-purified DNA was used for electroporation into *M. tuberculosis. Mycobacterium tuberculosis* strains were grown to an OD_580_ of ~0.4 to 1, washed, and resuspended in 10% glycerol to make electrocompetent cells as described in detail in (62). Bacteria were inoculated onto 7H11 agar with 50 µg/mL hygromycin as needed; a no-DNA control electroporation was done to control for spontaneously antibiotic-resistant mutants. Two weeks after plating, colonies were picked and inoculated into 200 µL 7H9c with antibiotics and then inoculated into 5-mL cultures for further analysis. Mutants were confirmed by PCR and sequence analysis.

### MymT luminescence from *M. tuberculosis* lysates

We adapted a previously reported protocol for measuring Cu(I)-thiolate core luminescence for use on filtered *M. tuberculosis* lysates (19). *M. tuberculosis* cultures were grown in Sauton minimal media to an OD_580_ = 0.3–0.5 and treated with *p*HBA to a final concentration of 1.2 mM as needed, and 50 µM CuSO_4_ 4 hours later. Twenty-four hours after Cu addition, 12 OD_580_ equivalent cell numbers were harvested by centrifugation and washed twice with buffer (10 mM HEPES, 150 mM NaCl pH 7.4). Bacteria were resuspended in 700 µL of the same buffer and lysed by bead beating as described for preparing lysates for immunoblotting. Lysates were then centrifuged for 7.5 minutes at 20,000 *g*, and the supernatants were passed through a 0.2-µ spin filter twice before application onto a Superose-6 10/300 GL column (Cytiva). Fractions were transferred to a UV-grade 96-well plate (Corning), and luminescence was measured with excitation at 280 nm, emission at 595 nm, and a cutoff of 325 nm.

For immunoblotting proteins in fractionated lysates, 200 µL of fractions corresponding to the three at the peak fluorescence collected using method above were stored at –20°C. Fractions were then thawed on ice, pooled, and concentrated in a 0.5-mL centrifugal filter (Amicon). Samples were boiled in SDS sample buffer for 10 minutes before separation on 15% SDS-PAGE gels and transferred onto nitrocellulose membranes. Polyclonal MymT antibodies used for immunoblotting were a kind gift from Ben Gold and Carl Nathan (19).

## SUPPLEMENTAL MATERIAL

Table S1 mbio.00363-23-s0001.xlsx Gene expression data of transcripts of *M. tuberculosis* treated with or without pHBA.

Table S2 mbio.00363-23-s0002.xlsx GO analysis of transcriptomics.

ASM does not own the copyrights to Supplemental Material that may be linked to, or accessed through, an article. The authors have granted ASM a non-exclusive, world-wide license to publish the Supplemental Material files. Please contact the corresponding author directly for reuse.

## References

[1] Nathan C , Shiloh MU . 2000. Reactive oxygen and nitrogen intermediates in the relationship between mammalian hosts and microbial pathogens. Proc Natl Acad Sci U S A 97:8841–8848. doi:10.1073/pnas.97.16.8841 10922044 PMC34021

[2] MacMicking JD , Nathan C , Hom G , Chartrain N , Fletcher DS , Trumbauer M , Stevens K , Xie QW , Sokol K , Hutchinson N , Chen H , Mudget JS . 1995. Altered responses to bacterial infection and endotoxic shock in mice lacking inducible nitric oxide synthase. Cell 81:641–650. doi:10.1016/0092-8674(95)90085-3 7538909

[3] Darwin KH , Ehrt S , Gutierrez-Ramos J-C , Weich N , Nathan CF . 2003. The proteasome of Mycobacterium tuberculosis is required for resistance to nitric oxide. Science 302:1963–1966. doi:10.1126/science.1091176 14671303

[4] Becker SH , Darwin KH . 2017. Bacterial proteasomes: mechanistic and functional insights. Microbiol Mol Biol Rev 81:e00036-16. doi:10.1128/MMBR.00036-16 PMC5312241 27974513

[5] Samanovic MI , Tu S , Novák O , Iyer LM , McAllister FE , Aravind L , Gygi SP , Hubbard SR , Strnad M , Darwin KH . 2015. Proteasomal control of cytokinin synthesis protects Mycobacterium tuberculosis against nitric oxide. Mol Cell 57:984–994. doi:10.1016/j.molcel.2015.01.024 25728768 PMC4369403

[6] Argueso CT , Ferreira FJ , Kieber JJ . 2009. Environmental perception avenues: the interaction of cytokinin and environmental response pathways. Plant Cell Environ 32:1147–1160. doi:10.1111/j.1365-3040.2009.01940.x 19183294

[7] Samanovic MI , Hsu H-C , Jones MB , Jones V , McNeil MR , Becker SH , Jordan AT , Strnad M , Xu C , Jackson M , Li H , Darwin KH . 2018. Cytokinin signaling in Mycobacterium tuberculosis. mBio 9:e00989-18. doi:10.1128/mBio.00989-18 29921668 PMC6016246

[8] Popelková H , Fraaije MW , Novák O , Frébortová J , Bilyeu KD , Frébort I . 2006. Kinetic and chemical analyses of the cytokinin dehydrogenase-catalysed reaction: correlations with the crystal structure. Biochem J 398:113–124. doi:10.1042/BJ20060280 16686601 PMC1525011

[9] Wagner D , Maser J , Lai B , Cai Z , Barry CE , Höner Zu Bentrup K , Russell DG , Bermudez LE . 2005. Elemental analysis of Mycobacterium avium-, Mycobacterium tuberculosis-, and Mycobacterium smegmatis-containing phagosomes indicates pathogen-induced microenvironments within the host cell’s endosomal system. J Immunol 174:1491–1500. doi:10.4049/jimmunol.174.3.1491 15661908

[10] White C , Lee J , Kambe T , Fritsche K , Petris MJ . 2009. A role for the ATP7A copper-transporting ATPase in macrophage bactericidal activity. J Biol Chem 284:33949–33956. doi:10.1074/jbc.M109.070201 19808669 PMC2797165

[11] Samanovic MI , Ding C , Thiele DJ , Darwin KH . 2012. Copper in microbial pathogenesis: meddling with the metal. Cell Host & Microbe 11:106–115. doi:10.1016/j.chom.2012.01.009 22341460 PMC3285254

[12] Wolschendorf F , Ackart D , Shrestha TB , Hascall-Dove L , Nolan S , Lamichhane G , Wang Y , Bossmann SH , Basaraba RJ , Niederweis M . 2011. Copper resistance is essential for virulence of Mycobacterium tuberculosis. Proc Natl Acad Sci U S A 108:1621–1626. doi:10.1073/pnas.1009261108 21205886 PMC3029754

[13] Liu T , Ramesh A , Ma Z , Ward SK , Zhang L , George GN , Talaat AM , Sacchettini JC , Giedroc DP . 2006. CsoR is a novel Mycobacterium tuberculosis copper-sensing transcriptional regulator. Nat Chem Biol 3:60–68. doi:10.1038/nchembio844 17143269

[14] Ward SK , Hoye EA , Talaat AM . 2008. The global responses of Mycobacterium tuberculosis to physiological levels of copper. J Bacteriol 190:2939–2946. doi:10.1128/JB.01847-07 18263720 PMC2293257

[15] Festa RA , Jones MB , Butler-Wu S , Sinsimer D , Gerads R , Bishai WR , Peterson SN , Darwin KH . 2011. A novel copper-responsive regulon in Mycobacterium tuberculosis. Mol Microbiol 79:133–148. doi:10.1111/j.1365-2958.2010.07431.x 21166899 PMC3052634

[16] Rowland JL , Niederweis M . 2013. A multicopper oxidase is required for copper resistance in Mycobacterium tuberculosis. J Bacteriol 195:3724–3733. doi:10.1128/JB.00546-13 23772064 PMC3754562

[17] Ward SK , Abomoelak B , Hoye EA , Steinberg H , Talaat AM . 2010. CtpV: a putative copper exporter required for full virulence of Mycobacterium tuberculosis. Mol Microbiol 77:1096–1110. doi:10.1111/j.1365-2958.2010.07273.x 20624225 PMC2965804

[18] Shi X , Festa RA , Ioerger TR , Butler-Wu S , Sacchettini JC , Darwin KH , Samanovic MI . 2014. The copper-responsive RicR regulon contributes to Mycobacterium tuberculosis virulence. mBio 5:e00876-13. doi:10.1128/mBio.00876-13 24549843 PMC3944814

[19] Gold B , Deng H , Bryk R , Vargas D , Eliezer D , Roberts J , Jiang X , Nathan C . 2008. Identification of a copper-binding metallothionein in pathogenic mycobacteria. Nat Chem Biol 4:609–616. doi:10.1038/nchembio.109 18724363 PMC2749609

[20] Bardarov S , Bardarov S , Pavelka MS , Sambandamurthy V , Larsen M , Tufariello J , Chan J , Hatfull G , Jacobs WR . 2002. Specialized transduction: an efficient method for generating marked and unmarked targeted gene disruptions in Mycobacterium tuberculosis, M. bovis BCG and M. smegmatis. Microbiology (Reading) 148:3007–3017. doi:10.1099/00221287-148-10-3007 12368434

[21] Ballister ER , Samanovic MI , Darwin KH . 2019. Mycobacterium tuberculosis Rv2700 contributes to cell envelope integrity and virulence. J Bacteriol 201:e00228-19. doi:10.1128/JB.00228-19 31285241 PMC6755743

[22] Marcus SA , Sidiropoulos SW , Steinberg H , Talaat AM . 2016. CsoR is essential for maintaining copper homeostasis in Mycobacterium tuberculosis. PLoS One 11:e0151816. doi:10.1371/journal.pone.0151816 26999439 PMC4801387

[23] Sato M , Bremner I . 1993. Oxygen free radicals and metallothionein. Free Radic Biol Med 14:325–337. doi:10.1016/0891-5849(93)90029-t 8458590

[24] LoPachin RM , Gavin T . 2014. Molecular mechanisms of aldehyde toxicity: a chemical perspective. Chem Res Toxicol 27:1081–1091. doi:10.1021/tx5001046 24911545 PMC4106693

[25] Beltramini M , Münger K , Germann UA , Lerch K . 1987. Luminescence emission from the Cu (I)-thiolate complex in metallothioneins. Experientia Suppl 52:237–241. doi:10.1007/978-3-0348-6784-9_17 2959510

[26] Darwin KH , Stanley SA . 2022. The aldehyde hypothesis: metabolic intermediates as antimicrobial effectors. Open Biol 12:220010. doi:10.1098/rsob.220010 35414258 PMC9006002

[27] Braverman J , Stanley SA . 2017. Nitric oxide modulates macrophage responses to Mycobacterium tuberculosis infection through activation of HIF-1α and repression of NF-κB. J Immunol 199:1805–1816. doi:10.4049/jimmunol.1700515 28754681 PMC5568107

[28] Braverman J , Sogi KM , Benjamin D , Nomura DK , Stanley SA . 2016. HIF-1α is an essential mediator of IFN-γ-dependent immunity to Mycobacterium tuberculosis. J Immunol 197:1287–1297. doi:10.4049/jimmunol.1600266 27430718 PMC4976004

[29] Gleeson LE , Sheedy FJ , Palsson-McDermott EM , Triglia D , O’Leary SM , O’Sullivan MP , O’Neill LAJ , Keane J . 2016. Cutting edge: Mycobacterium tuberculosis induces aerobic glycolysis in human alveolar macrophages that is required for control of intracellular bacillary replication. J Immunol 196:2444–2449. doi:10.4049/jimmunol.1501612 26873991

[30] Escoll P , Song O-R , Viana F , Steiner B , Lagache T , Olivo-Marin J-C , Impens F , Brodin P , Hilbi H , Buchrieser C . 2017. Legionella pneumophila modulates mitochondrial dynamics to trigger metabolic repurposing of infected macrophages. Cell Host Microbe 22:302–316. doi:10.1016/j.chom.2017.07.020 28867389

[31] Rachman H , Kim N , Ulrichs T , Baumann S , Pradl L , Nasser Eddine A , Bild M , Rother M , Kuban R-J , Lee JS , Hurwitz R , Brinkmann V , Kosmiadi GA , Kaufmann SHE . 2006. Critical role of methylglyoxal and AGE in mycobacteria-induced macrophage apoptosis and activation. PLoS One 1:e29. doi:10.1371/journal.pone.0000029 17183656 PMC1762319

[32] Safi H , Gopal P , Lingaraju S , Ma S , Levine C , Dartois V , Yee M , Li L , Blanc L , Ho Liang H-P , Husain S , Hoque M , Soteropoulos P , Rustad T , Sherman DR , Dick T , Alland D . 2019. Phase variation in Mycobacterium tuberculosis glpK produces transiently heritable drug tolerance. Proc Natl Acad Sci U S A 116:19665–19674. doi:10.1073/pnas.1907631116 31488707 PMC6765255

[33] Bellerose MM , Baek S-H , Huang C-C , Moss CE , Koh E-I , Proulx MK , Smith CM , Baker RE , Lee JS , Eum S , Shin SJ , Cho S-N , Murray M , Sassetti CM . 2019. Common variants in the glycerol kinase gene reduce tuberculosis drug efficacy. mBio 10:e00663-19. doi:10.1128/mBio.00663-19 31363023 PMC6667613

[34] Vargas R , Farhat MR . 2020. Antibiotic treatment and selection for glpK mutations in patients with active tuberculosis disease. Proc Natl Acad Sci U S A 117:3910–3912. doi:10.1073/pnas.1920788117 32075922 PMC7049102

[35] Maciag A , Dainese E , Rodriguez GM , Milano A , Provvedi R , Pasca MR , Smith I , Palù G , Riccardi G , Manganelli R . 2007. Global analysis of the Mycobacterium tuberculosis zur (FurB) regulon. J Bacteriol 189:730–740. doi:10.1128/JB.01190-06 17098899 PMC1797298

[36] Rodriguez GM , Voskuil MI , Gold B , Schoolnik GK , Smith I . 2002. IdeR, an essential gene in Mycobacterium tuberculosis: role of ideR in iron-dependent gene expression, iron metabolism, and oxidative stress response. Infect Immun 70:3371–3381. doi:10.1128/IAI.70.7.3371-3381.2002 12065475 PMC128082

[37] Chownk M , Sharma A , Singh K , Kaur J . 2017. MesT, a unique epoxide hydrolase, is essential for optimal growth of Mycobacterium tuberculosis in the presence of styrene oxide. Future Microbiol 12:527–546. doi:10.2217/fmb-2016-0206 28492351

[38] Madacki J , Laval F , Grzegorzewicz A , Lemassu A , Záhorszká M , Arand M , McNeil M , Daffé M , Jackson M , Lanéelle MA , Korduláková J . 2018. Impact of the epoxide hydrolase EphD on the metabolism of mycolic acids in mycobacteria. J Biol Chem 293:5172–5184. doi:10.1074/jbc.RA117.000246 29472294 PMC5892587

[39] Fang H , Yu D , Hong Y , Zhou X , Li C , Sun B . 2013. The LuxR family regulator Rv0195 modulates Mycobacterium tuberculosis dormancy and virulence. Tuberculosis (Edinb) 93:425–431. doi:10.1016/j.tube.2013.04.005 23673208

[40] Pinto R , Tang QX , Britton WJ , Leyh TS , Triccas JA . 2004. The Mycobacterium tuberculosis cysD and cysNC genes form a stress-induced operon that encodes a tri-functional sulfate-activating complex. Microbiology (Reading) 150:1681–1686. doi:10.1099/mic.0.26894-0 15184554

[41] Rustad TR , Minch KJ , Ma S , Winkler JK , Hobbs S , Hickey M , Brabant W , Turkarslan S , Price ND , Baliga NS , Sherman DR . 2014. Mapping and manipulating the Mycobacterium tuberculosis transcriptome using a transcription factor overexpression-derived regulatory network. Genome Biol 15:502. doi:10.1186/PREACCEPT-1701638048134699 25380655 PMC4249609

[42] Busenlehner LS , Pennella MA , Giedroc DP . 2003. The SmtB/ArsR family of metalloregulatory transcriptional repressors: structural insights into prokaryotic metal resistance. FEMS Microbiol Rev 27:131–143. doi:10.1016/S0168-6445(03)00054-8 12829264

[43] Chawla M , Parikh P , Saxena A , Munshi M , Mehta M , Mai D , Srivastava AK , Narasimhulu KV , Redding KE , Vashi N , Kumar D , Steyn AJC , Singh A . 2012. Mycobacterium tuberculosis WhiB4 regulates oxidative stress response to modulate survival and dissemination in vivo. Mol Microbiol 85:1148–1165. doi:10.1111/j.1365-2958.2012.08165.x 22780904 PMC3438311

[44] Buglino JA , Sankhe GD , Lazar N , Bean JM , Glickman MS . 2021. Integrated sensing of host stresses by inhibition of a cytoplasmic two-component system controls M. Tuberculosis acute lung infection. Elife 10:e65351. doi:10.7554/eLife.65351 34003742 PMC8131098

[45] Wang L , Zhu M , Zhang Q , Zhang X , Yang P , Liu Z , Deng Y , Zhu Y , Huang X , Han L , Li S , He J . 2017. Diisonitrile natural product SF2768 functions as a chalkophore that mediates copper acquisition in Streptomyces thioluteus. ACS Chem Biol 12:3067–3075. doi:10.1021/acschembio.7b00897 29131568

[46] Xu Y , Tan DS . 2019. Total synthesis of the bacterial diisonitrile chalkophore SF2768. Org Lett 21:8731–8735. doi:10.1021/acs.orglett.9b03348 31633364 PMC6905096

[47] Buglino JA , Ozakman Y , Xu Y , Chowdhury F , Tan DS , Glickman MS . 2022. Diisonitrile lipopeptides mediate resistance to copper starvation in pathogenic mycobacteria. mBio 13:e0251322. doi:10.1128/mbio.02513-22 36197089 PMC9600254

[48] Thornalley PJ . 1988. Modification of the glyoxalase system in human red blood cells by glucose in vitro. Biochem J 254:751–755. doi:10.1042/bj2540751 3196289 PMC1135147

[49] Phillips SA , Thornalley PJ . 1993. The formation of methylglyoxal from triose phosphates. investigation using a specific assay for methylglyoxal. Eur J Biochem 212:101–105. doi:10.1111/j.1432-1033.1993.tb17638.x 8444148

[50] Mazurek S , Boschek CB , Hugo F , Eigenbrodt E . 2005. Pyruvate kinase type M2 and its role in tumor growth and spreading. Semin Cancer Biol 15:300–308. doi:10.1016/j.semcancer.2005.04.009 15908230

[51] Fan J , Kamphorst JJ , Mathew R , Chung MK , White E , Shlomi T , Rabinowitz JD . 2013. Glutamine-driven oxidative phosphorylation is a major ATP source in transformed mammalian cells in both normoxia and hypoxia. Mol Syst Biol 9:712. doi:10.1038/msb.2013.65 24301801 PMC3882799

[52] Shi L , Salamon H , Eugenin EA , Pine R , Cooper A , Gennaro ML . 2015. Infection with Mycobacterium tuberculosis induces the Warburg effect in mouse lungs. Sci Rep 5:18176. doi:10.1038/srep18176 26658723 PMC4674750

[53] Anaya-Sanchez A , Feng Y , Berude JC , Portnoy DA . 2021. Detoxification of methylglyoxal by the glyoxalase system is required for glutathione availability and virulence activation in Listeria monocytogenes. PLoS Pathog 17:e1009819. doi:10.1371/journal.ppat.1009819 34407151 PMC8372916

[54] Li H , Durbin R . 2009. Fast and accurate short read alignment with Burrows–Wheeler transform. Bioinformatics 25:1754–1760. doi:10.1093/bioinformatics/btp324 19451168 PMC2705234

[55] Li H , Handsaker B , Wysoker A , Fennell T , Ruan J , Homer N , Marth G , Abecasis G , Durbin R , 1000 Genome Project Data Processing Subgroup . 2009. The sequence alignment/map format and SAMtools. Bioinformatics 25:2078–2079. doi:10.1093/bioinformatics/btp352 19505943 PMC2723002

[56] Liao Y , Smyth GK , Shi W . 2014. FeatureCounts: an efficient general purpose program for assigning sequence reads to genomic features. Bioinformatics 30:923–930. doi:10.1093/bioinformatics/btt656 24227677

[57] Team RC . 2022. R: a language and environment for statistical computing. Vienna, Austria. Available from: https://www.R-project.org/

[58] Love MI , Huber W , Anders S . 2014. Moderated estimation of fold change and dispersion for RNA-Seq data with DESeq2. Genome Biol 15:550. doi:10.1186/s13059-014-0550-8 25516281 PMC4302049

[59] Bedre R . 2020. Reneshbedre/Bioinfokit: bioinformatics data analysis and visualization toolkit. doi:10.5281/zenodo.3965241

[60] Gallagher S , Winston SE , Fuller SA , Hurrell JGR . 2004. Immunoblotting and immunodetection. Curr Protoc Mol Biol 66:10.8.1-10.8.24. doi:10.1002/0471142727.mb1008s66 18265338

[61] Jastrab JB , Wang T , Murphy JP , Bai L , Hu K , Merkx R , Huang J , Chatterjee C , Ovaa H , Gygi SP , Li H , Darwin KH . 2015. An adenosine triphosphate-independent proteasome activator contributes to the virulence of Mycobacterium tuberculosis. Proc Natl Acad Sci U S A 112:E1763–E1772. doi:10.1073/pnas.1423319112 25831519 PMC4394314

[62] Hatfull GF , Jacobs WR . 2000. Molecular Genetics of mycobacteria. ASM Press, Washington, DC.

